# Numerical and structural aberrations in advanced neuroblastoma tumours by CGH analysis; survival correlates with chromosome 17 status

**DOI:** 10.1054/bjoc.2000.1432

**Published:** 2000-10-26

**Authors:** C Lo Cunsolo, M P Bicocchi, A R Petti, G P Tonini

**Affiliations:** Solid Tumor Biology Unit, Advanced Biotechnology Center; Department of Hematology/Oncology, G. Gaslini Children's Hospital; Department of Statistics, University of Genoa; Laboratory of Population Genetics, National Institute for Cancer Research (IST), Genoa, Italy

**Keywords:** neuroblastoma, comparative genomic hybridization, chromosome 17

## Abstract

Rapid tumour progression in neuroblastoma is associated with *MYCN* amplification, deletion of the short arm of chromosome 1 and gain of 17q. However, patients with advanced disease without *MYCN* amplification and/or 1p deletion have a very poor outcome too, which suggests other genetic defects may predict an unfavourable prognosis. We employed CGH to study 22 tumours of patients at stages 3 and 4 over one year of age (6 and 16 cases respectively). Patients were divided in groups (A) long-term survivors and (B) short-term survivors. CGH showed a total of 226 chromosome imbalances (110 in group A and 116 in group B). The neuroblastoma cells of long-term survivors showed a preponderance of numerical aberrations (54%*vs* 43%); particularly gains of entire chromosomes 1 (*P*< 0.03), 7 (*P*< 0.04) and 19 (*P*< 0.05). An extra copy of 17 was detected in 6/8 (75%) samples of group A and only 1/14 (7%) samples of group B (*P*< 0.002). Conversely, tumours of patients who died from disease progression displayed a higher frequency of structural abnormalities (43%*vs* 35%), including loss of 1p, 9p, 11q, 15q and 18q and gain of 12q, although the difference was not significant (*P*= 0.24). Unbalanced gain of 17q was detected in 8/14 (57%) tumours of group B and only 1/8 (13%) tumours of group A (*P*< 0.05). The peculiar genetic difference observed in the tumours of long and short-term survivors may have prognostic relevance. © 2000 Cancer Research Campaign


British Journal of Cancer (2000) 83(10), 1295–1300
© 2000 Cancer Research Campaign
doi: 10.1054/ bjoc.2000.1432, available online at http://www.idealibrary.com on IDEAL®

### Numerical and structural aberrations in advanced neuroblastoma tumours by CGH analysis; survival correlates with chromosome 17 status

**C Lo Cunsolo[1], MP Bicocchi[2], AR Petti[3] and GP Tonini[1,4]**

[1] Solid Tumor Biology Unit, Advanced Biotechnology Center; [2]Department of Hematology/Oncology, G. Gaslini Children's Hospital; [3]Department of Statistics, University of Genoa; [4]Laboratory of Population Genetics, National Institute for Cancer Research (IST); Genoa, Italy

**Summary** Rapid tumour progression in neuroblastoma is associated with *MYCN* amplification, deletion of the short arm of chromosome 1 and gain of 17q. However, patients with advanced disease without *MYCN* amplification and/or 1p deletion have a very poor outcome too, which suggests other genetic defects may predict an unfavourable prognosis. We employed CGH to study 22 tumours of patients at stages 3 and 4 over one year of age (6 and 16 cases respectively). Patients were divided in groups (A) long-term survivors and (B) short-term survivors. CGH showed a total of 226 chromosome imbalances (110 in group A and 116 in group B). The neuroblastoma cells of long-term survivors showed a preponderance of numerical aberrations (54% *vs* 43%); particularly gains of entire chromosomes 1 ($P < 0.03$), 7 ($P < 0.04$) and 19 ($P < 0.05$). An extra copy of 17 was detected in 6/8 (75%) samples of group A and only 1/14 (7%) samples of group B ($P < 0.002$). Conversely, tumours of patients who died from disease progression displayed a higher frequency of structural abnormalities (43% *vs* 35%), including loss of 1p, 9p, 11q, 15q and 18q and gain of 12q, although the difference was not significant ($P = 0.24$). Unbalanced gain of 17q was detected in 8/14 (57%) tumours of group B and only 1/8 (13%) tumours of group A ($P < 0.05$). The peculiar genetic difference observed in the tumours of long and short-term survivors may have prognostic relevance. © 2000 Cancer Research Campaign

**Keywords:** neuroblastoma; comparative genomic hybridization; chromosome 17

Neuroblastoma is a childhood tumour characterized by genetic heterogeneity and non-random chromosome defects associated with rapid tumour progression. *MYCN* gene amplification (Seeger et al, 1985), deletion of chromosome 1p36 (Caron et al, 1996) and gain of 17q (Bown et al, 1999) are associated with a poor prognosis. Over the last few years the Comparative Genomic Hybridization (CGH) technique has been performed with success to detect genomic imbalances in both frozen and paraffin-embedded tumour tissue. In neuroblastoma CGH has revealed the presence of multiple genetic defects, including losses of chromosomes 4p, 9p, 11q, 14q and gains of 17q, 4q and 6p (Brinkschmidt et al, 1997; Lastowska et al, 1997; Plantaz et al, 1997; Vandesompele et al, 1998).

The outcome of patients with localized neuroblastoma is generally good, but patients with metastatic disease have a very poor survival rate despite the newest therapies. The better outcome of the former group probably depends on the different genetic and molecular alterations present in their tumours. Furthermore, a subset of patients with advanced disease has a longer survival rate, showing complete remission or a stable disease. We hypothesized that the tumours of this subset have distinct features, allowing them to survive.

We examined 22 samples of neuroblastoma by CGH to investigate the genetic aspects of the tumours of patients with advanced neuroblastoma and long *vs* short-term survival. According to our results the tumours of patients who died from disease progression show a significantly higher number of structural chromosome abnormalities and gain of 17q, in comparison to the numerical aberrations and 17 trisomy that characterize the tumour of long-term survivors. The particular genetic pattern observed in the latter may be useful for the prediction of a better outcome for patients with advanced neuroblastoma.

*Received 5 January 2000*
*Revised 12 June 2000*
*Accepted 16 June 2000*

*Correspondence to:* GP Tonini

### MATERIALS AND METHODS

### Patients

22 tumour cell samples collected at time of surgery before any treatment and stored in the Italian Neuroblastoma Tissue Bank were used for CGH analysis. All patients were over 1 year old at time of diagnosis, and 21 out 22 had an abdominal primary. Patients were staged according to INSS (Brodeur et al, 1993) and tumours classified according to the Shimada criteria (1984). Patients were treated following the current protocols by the Italian Cooperative Neuroblastoma Study Group. The samples included in the study derived from 6 patients at stage 3 and 16 at stage 4. Patients were selected according to their status at time of the analysis (Table 1). All patients of group A were alive with at least a 3-year follow up. In group B, all except patient 21 have a shorter survival.

### Tumour samples

High molecular weight DNA was isolated from primary tumours or bone marrow aspirates at time of diagnosis and from leukocytes

**Table 1** Clinical data and follow up of 22 patients with advanced neuroblastomas, *MYCN* gene amplification, chromosome 1 and 17 status in tumour samples

| Group A Case | Stage | Age at diagnosis[a] | Follow up[a] | Chr 2 by CGH/*MYCN* by FISH & SB | Chr 1 by CGH/1p by FISH or LOH | Chr. 17 by CGH |
|---|---|---|---|---|---|---|
| 1 | 3 | 22 | 72,CR | normal/normal | +1/n.d. | +17 |
| 2 | 4 | 22 | 60,CR | gain/normal | normal/n.d. | +17 |
| 3 | 4 | 27 | 72,CR | normal/normal | +1/n.d. | normal |
| 4 | 4 | 31 | 48,CR | normal/normal | +1/normal | +17 |
| 5 | 4 | 32 | 60,CR | gain/ampl | 1q loss/n.d. | +17q |
| 6 | 4 | 79 | 60,AWD | gain/normal | +1/normal | +17 |
| 7 | 4 | 86 | 48,CR | normal/normal | 1q loss/normal | +17 |
| 8 | 4 | 101 | 48,AWD | normal/normal | +1/n.d. | +17 |
| **Group B** | | | | | | |
| 9 | 3 | 15 | 13,DWD | gain/normal | 1p loss/deleted | +17q |
| 10 | 3 | 23 | 20,DWD | gain/ampl | 1p loss/deleted | normal |
| 11 | 3 | 28 | 13,DWD | gain/ampl | normal/deleted | normal |
| 12 | 3 | 50 | 18,DWD | normal/normal | normal/normal | +17q |
| 13 | 3 | 52 | 6,DWD | normal/normal | normal/normal | +17q |
| 14 | 4 | 12 | 1,TOX | gain/normal | normal/normal | +17q |
| 15 | 4 | 26 | 9,DWD | normal/normal | normal/normal | +17q |
| 16 | 4 | 26 | 17,DWD | gain/ampl | 1q gain/deleted | +17q |
| 17 | 4 | 29 | 30,DWD | normal/normal | normal/normal | +17 |
| 18 | 4 | 32 | 11,DWD | normal/normal | normal/normal | 17qdel |
| 19 | 4 | 49 | 20,DWD | normal/normal | +1/normal | +17q |
| 20 | 4 | 58 | 1,DWD | gain/normal | 1p loss/deleted | +17q |
| 21 | 4 | 132 | 53,DWD | normal/normal | 1p loss/deleted | normal |
| 22 | 4 | 138 | 9,DWD | normal/ampl | normal/deleted | normal |

[a] Age at diagnosis and follow up are expressed in months, CR: complete remission; AWD: alive with disease; DWD: died with disease, TOX: died from toxicity; n.d.: not done; n.i.: not informative; SB: Southern blot of normal male donors (Perri et al, 1996). Tumour cell content was more than 90% in all samples.

### Comparative genomic hybridization

CGH was performed as described by Kallioniemi et al (1992). Metaphases were prepared from phytohaemagglutinin-stimulated peripheral blood lymphocytes of normal healthy male donors according to standard procedures. Digital images were collected through a computer-controlled Nikon E800 fluorescence microscope (Nikon, Italy) connected to a Cytovision Workstation (Applied Imaging, Santa Clara, CA). Three-colour images representing tumour (green), reference DNA (red) and DAPl counterstain (blue) were captured for each metaphase and analysed as separate grey-scale images. Chromosomes were identified by DAPI; green and red fluorescence intensities were then scored along the vertical medial axis of each chromosome. For evaluation of CGH data average ratio profiles with fixed limits at 1.25 and 0.75 (standard deviation limits: 95%) and individual ratio profiles were analysed. Chromosomes X and Y were excluded from the analysis. CGH controls were performed by matching two normal donors' purified DNAs.

### Fluorescence in situ hybridization (FISH) and loss of heterozygosity (LOH) for chromosome 1p36

Chromosome 1p deletion was evaluated by two-colour FISH on tumour cell interphase nuclei as previously described (Lo Cunsolo et al, 1999). Probes pI-79 (locus D1Z2 at 1pter) and QC (chromosome 1 centromere) were used. LOH for 1p36 was studied by the PCR method using the primer sets for D1S80 and D1S76 loci according to Peter et al (1992).

### MYCN gene amplification

*MYCN* amplification was determined by Southern blot as previously described (Tonini et al, 1997) and, when possible, by double colour FISH using the NB-19-21 and D2Z (Oncor, Heidelberg, Germany) chromosome 2 centromeric probes.

### Statistic analysis

$\chi^2$ test was employed to compare gains of whole chromosomes in tumours of living and dead patients. The differences in the frequency of chromosome abnormality found in tumour samples were tested by Fisher's exact test.

### RESULTS

We studied by CGH 22 tumour samples of patients with advanced neuroblastoma, including long-term survival patients (group A) and patients who died from disease progression (group B). The median follow up was 60 (48–72) months for group A and 27 (1–53) months for group B. Six out of eight patients of group A, up until now, are in complete remission; the remaining 2 are alive with stable disease and do not require further treatment. All patients of group B (except case no. 14) died from disease progression. Patient no. 14 died from toxicity (Table 1).

We detected a total of 226 chromosome imbalances, 110 in group A and 116 in group B. Group A shows a higher frequency of numerical aberrations in term of gains and losses (54% *vs* 43%). Whole chromosome gains were more prevalent in group A (37%) compared to group B (27%) ($\chi^2$ = 2.89; $P$ < 0.09). Group A shows 35% of structural abnormalities (gene amplification excluded) compared with 43% observed in group B ($\chi^2$ = 1.38; $P$ < 0.24).

 *© 2000 Cancer Research Campaign*

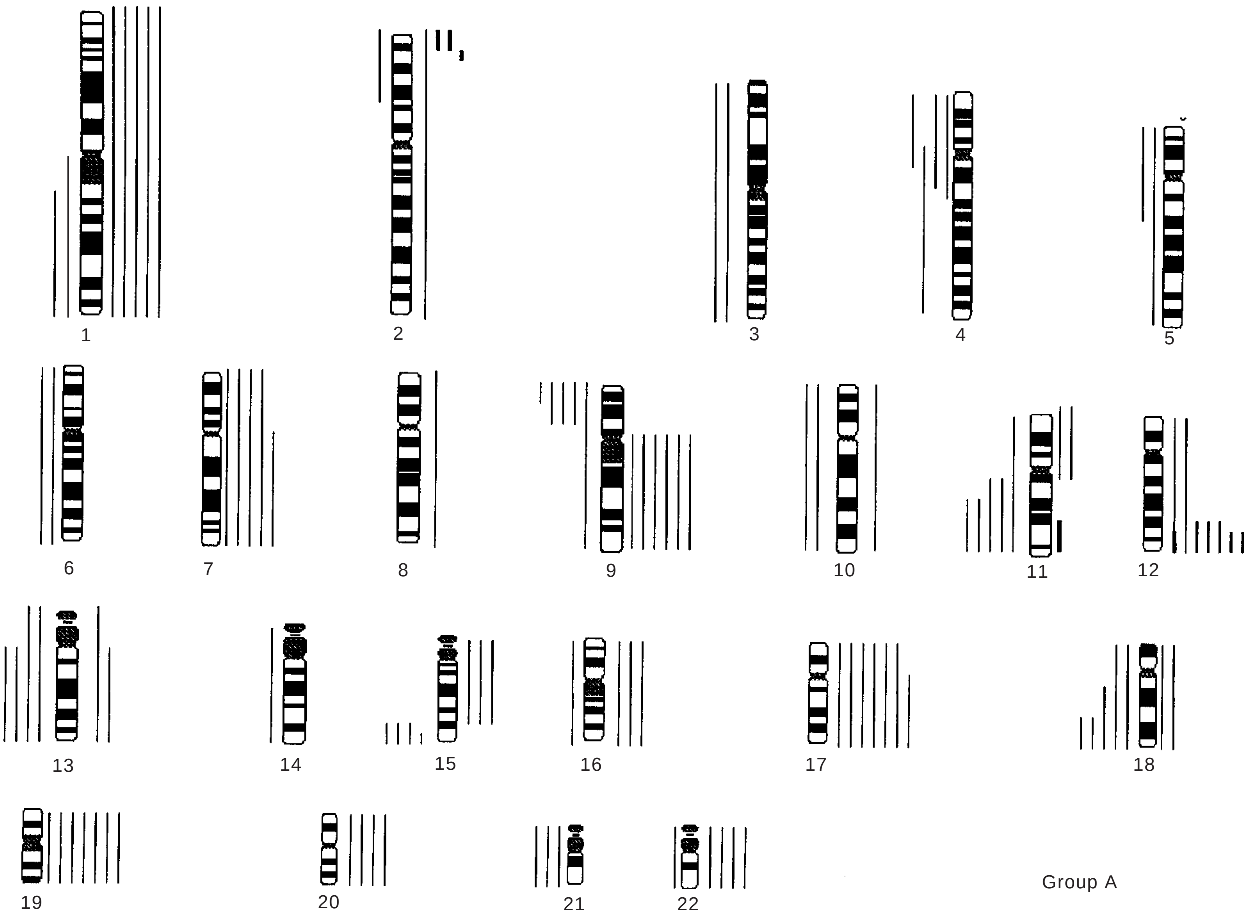

**Figure 1** Ideogram of DNA imbalances in 22 samples of neuroblastoma tumours of group A (top panel) and group B (bottom panel) patients (see Table 1 for details). Lines on the left side of the ideograms indicate under-representations, lines and bars on the right side indicate over-representation and gene amplification respectively

Results of chromosome imbalances are reported in the ideogram in Figure 1.

### Whole chromosome gains and losses

In group A extra copies of chromosomes 1, 7, 17 and 19 were observed in 5/8, 4/8, 6/8 and 7/8 cases (63%, 50%, 75% and 88% respectively). In group B additional chromosomes 1, 7, 17 and 19 were present in 2/14, 1/14, 1/14 and 6/14 cases (14%, 7%, 7% and 43%) (Figure 1). A statistically significant difference for chromosome gains 1 ($P < 0.03$), 7 ($P < 0.04$), 19 ($P < 0.05$) and 17 ($P < 0.002$) stood out between group A and B tumors. These differences are shown in Figures 2A, B.

### Partial chromosome gains and losses

Additional chromosome 17q material was found in 1/8 (13%) samples of group A and 8/14 (57%) samples of group B. In the case of group A and in 3/8 cases of group B gain of 17q includes the region 17q11.2→qter whereas 5/8 cases of group B shows gain from 17q21 to qter (Figure 1). Chromosome 17q was significantly gained in the tumours of patients who died from disease progression ($P < 0.05$). The comparison between chromosome 17 and 17q gains in living and dead patients is presented in Figure 2B.

The 2p23–24 chromosome region was amplified in 9 tumours. Three cases belonged to group A patients and 6 to group B ones. Since this region includes the locus for *MYCN* gene, we performed Southern blot and/or FISH analysis to verify the presence of *MYCN* amplification. Four tumours (cases 5, 10, 11, and 16) showed gain of 2p24 region and *MYCN* amplification; in cases 2, 6, 9, 14 and 20 an excess of green signal at 2p24 did not correspond to *MYCN* amplification. Case 22 did not show imbalance at this region, but *MYCN* amplification was present, as detected by Southern blot.

Loss of chromosome 1p was detected in 7 tumours of group B but not in those of group A (Figure 1). Four cases of group B (9, 10, 20, 21) showed chromosome 1p deletion which was readily evident by CGH, whereas three additional cases (11, 16, 22) were detected by FISH and not by CGH (Table 1). This discrepancy likely reflects the intrinsic limitations of CGH, which can only detect deletions larger than 10 Mb in size (Van Gele et al, 1997; Van Roy et al, 1997). When constitutional DNA was available from patients, FISH results were confirmed by LOH.

Loss of chromosome 9p was found in 4/8 (50%) cases of group A and 2/14 (14%) cases of group B. Chromosome 11q was lost in 4/8 (50%) tumours of group A and 2/14 (14%) tumours of group B.

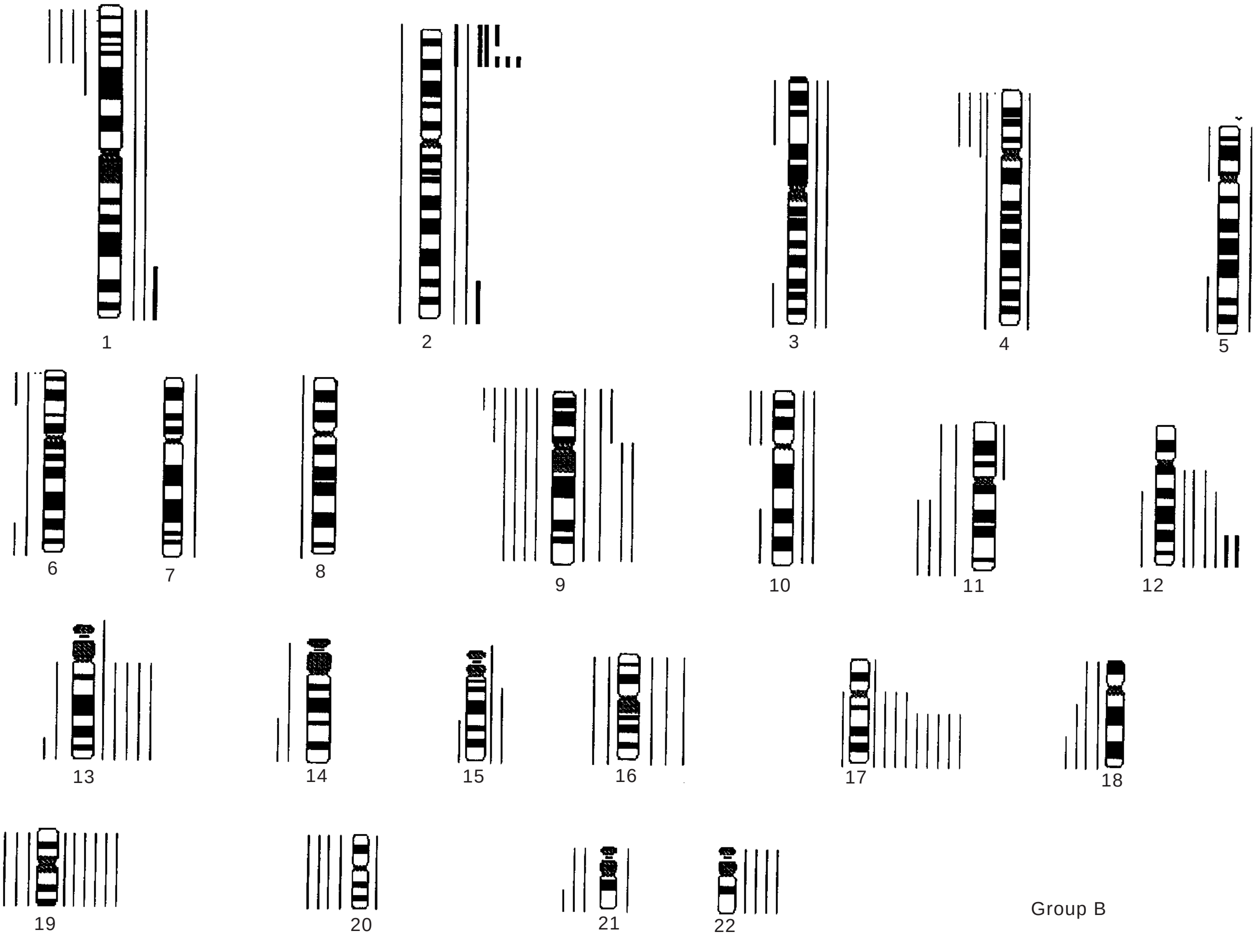

**Figure 1** continued.

### DISCUSSION

In the present report we show that tumours of surviving patients with advanced neuroblastoma are frequently characterized by numerical chromosome aberrations, whereas tumours of children who died from disease progression have more structural abnormalities, including gain of 17q. Particularly, we observed that gain of 17q was present in 8/14 (57%) tumours of group B and only 1/8 (13%) tumours of group A, which suggests that this chromosome plays an important role in tumour growth. On a total of 9 cases that show 17q gain, 4 cases belonging to group B display a gain that includes the region 17q11.2→qter and 5 the region q21→qter. The latter region has been frequently involved in the unbalanced translocation t(1p;17q) found in neuroblastoma cell lines (Lastowska et al, 1998). Indeed, in both neuroblastoma cell lines and tumours it has been shown that chromosome 17q can translocate to several different chromosomes, thus leading to remarkably complex translocations (Meddeb et al, 1996; Van Roy et al; 1997; Lastowska et al, 1998).

In the present work, gains of an entire chromosome 17 were detected in 75% of surviving children's tumours. We hypothesize that the additional copy of chromosome 17 derives from an abnormal mitosis or from the non-disjunction of chromosomes during the mitotic process. In either cases the additional copy of 17 should not alter the function of a specific gene enclosed on this chromosome. Our data suggest that the extra copy of chromosome 17 does not contribute to tumour aggressiveness. Conversely, the presence of +17q in group B appeared to be associated with a poor outcome and was not significantly associated with *MYCN* amplification or 1p deletion. Patient no. 5 (group A) represents an exception, since he is still alive and in complete remission, although his tumour shows unfavourable markers such as *MYCN* amplification and +17q. Bown et al (1999) have observed a strong association between *MYCN* amplification, chromosome 1p deletion and gain of 17q. Probably, the discrepancy between Bown's data and ours is caused by the limited number of samples that we analysed. At any rate, our results, together with the data reported by Bown et al (1999), further support a role of chromosome 17q in the development and progression of neuroblastoma. Finally, our analysis clearly defines a subset of longer-term survival patients whose tumours do not show gross 17q abnormality. This finding may contribute to identifying patients with advanced disease and a more favourable outcome.

A gain at the 2p24 was found in 5 tumours by CGH analysis without a *MYCN* gene amplification. In this region is mapped the *DDXI* gene; *DDXI* is far from *MYCN* gene of 400 kbp and has been found co-amplified in human neuroblastoma cell lines and tumours (Amler et al, 1996; George et al, 1997). Although, *DDXI* has been found, until now, co-amplified with *MYCN* (George et al, 1997) we may argue that gain at 2p24 region could be associated

 © 2000 Cancer Research Campaign

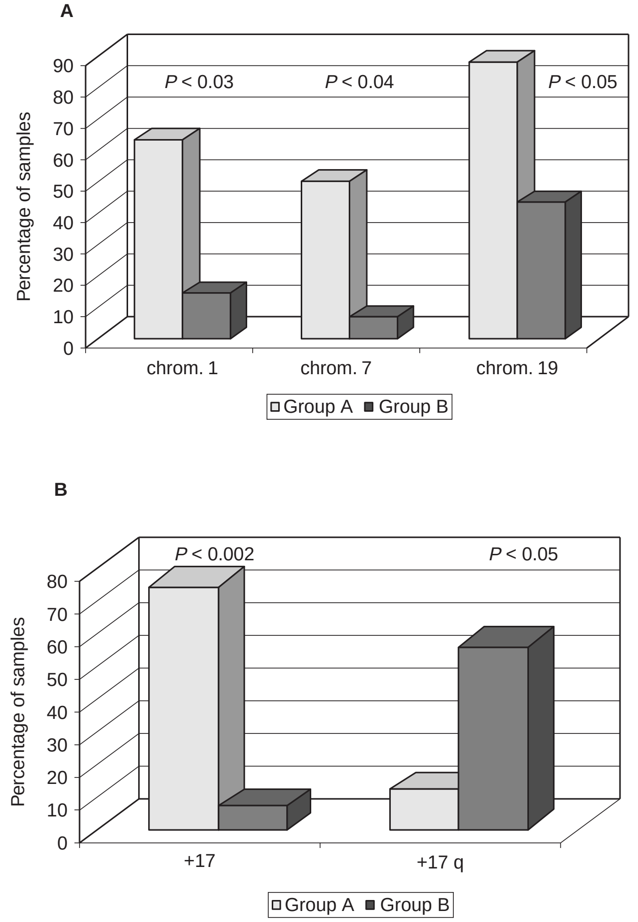

**Figure 2** (**A**) distribution of gains of whole chromosomes 1, 7 and 19 in tumours of long (group A) and short-term (group B) survival patients. Tumours of survived patients significantly gain an extra copy of 1 ($P < 0.03$), 7 ($P < 0.04$) and 19 ($P < 0.05$). (**B**) Distribution of gains of chromosome 17 and 17q in tumours of long (group A) and short-term (group B) survival patients. Tumours of survived patients significantly gain a whole chromosome 17 ($P < 0.002$), whereas tumours of patients who died from disease progression gain 17q ($P < 0.05$)

with the presence of *DDXI* gene extracopies or other amplifed DNA sequences (Wimmer et al, 1999).

Losses of 9p were detected in 50% of the tumours of group A and in 14% of group B, which confirms previous observations on the defect of this chromosome in advanced neuroblastoma (Takita et al, 1995; Iolascon et al, 1998). However, a higher frequency of 9p deletion in the tumours of group A suggests that $p16^{INK4A}$ gene, which usually shows allelic loss in 9p-deleted neuroblastomas, is not essential to tumour progression but participate only in the deregulation of cell cycle.

Hence, tumours of patients with advanced neuroblastoma seem to be characterized by non-random primary chromosome alterations such as *MYCN* amplification, 1p deletion and gain of 17q, along with secondary non-random abnormalities such as del(11q), del(9p) and gain of 12q. The role of chromosome 18q was already reported by Takita et al (1995), but in our cases loss of 18q was found both in group A and B. Gain of an entire chromosome 19 was observed in both groups. No rearrangements of this chromosome were detected, so its role in tumour progression still remains unclear.

Finally, it is interesting to note that the genetic features of the tumours of surviving patients are very similar to those of children with localized disease retaining additional copies of several chromosomes (Brinkschmidt et al, 1997; Plantaz et al, 1997; Vandesompele et al, 1998). This suggests that an extra copy of an entire chromosome in both localized and disseminated neuroblastoma characterizes a less aggressive tumour phenotype. Genome analysis by CGH may be considered a suitable tool to screen primary tumours at the onset of the disease in order to identify high-risk patients with advanced neuroblastoma.

### ACKNOWLEDGEMENTS

This work was supported by AIRC (Associazione Italiana per la Ricerca sul Cancro) and Associazione Italiana per la Lotta al Neuroblastoma. We are grateful to surgeons, clinicians and pathologists of the Italian Cooperative Group for Neuroblastoma and to AIEOP (Associazione Italiana di Ematologia e Oncologia Pediatrica) for providing tumour samples. We also wish to thank Prof GM Brodeur for reviewing and discussing the manuscript and Dr Chiara Perfumo for technical support. Dr F Alt (Columbia University, NY, USA) kindly provided the NB-19-21 probe. We also wish to thank the Ordine dei SS Maurizio e Lazzaro, Genova, for the donation of the Nikon microscope.

### REFERENCES

Amler LC, Schurmann J and Schwab M (1996) The *DDXI* gene maps within 400 kbp 5′ to *MYCN* and is frequently coamplified in human neuroblastoma. *Genes, Chromosom & Cancer* **15**: 134–137

Brinkschmidt C, Christiansen H, Terpe HJ, Simon R, Boecker W, Lampert F and Stoerkel S (1997) Comparative genomic hybridization (CGH) analysis of neuroblastomas – an important methodological approach in paediatric tumour pathology. *J Pathol* **181**: 394–400

Bown N, Cotterill S, Lastowska M, O'Neill S, Pearson ADJ, Plantaz D, Meddeb M, Danglot G, Brinkschidt C, Christiansen H, Laureys G and Speleman F (1999) Gain of chromosome arm 17q and adverse outcome in patients with neuroblastoma. *N Engl J Med* **340**: 1954–1961

Brodeur GM, Pritchard J, Berthold F, Carlsen NTL, Castel V, Castleberry RP, De Bernardi B, Evans AE, Favrot M, Hedborg F, Kaneko M, Kemshead J, Lampert F, Lee REJ, Look, T, Pearson ADJ, Philip T, Roald B, Sawada T, Seeger RC, Tsuchida Y and Vaute PA (1993) Revision of the international criteria for neuroblastoma diagnosis, staging and response to treatment. *J Clin Oncol* **11**: 1466–1477

Caron H, van Sluis P, de Kraker J, Bokkerink J, Egeler M, Laureys G, Slater R, Westerveld A, Voute PA and Versteeg R (1996) Allelic loss of chromosome 1p as a predictor of unfavorable outcome in patients with neuroblastoma. *N Engl J Med* **25**: 225–230

George RE, Kenyon AG, McGuckin AG, Kohl N, Kogner P, Christiansen H, Pearson ADJ and Lunec J (1997) Analysis of candidate gene co-amplified with *MYCN* in neuroblastoma. *Eur J Cancer* **12**: 2037–2042

Iolascon A, Giordani L, Tonini GP, Lo Cunsolo C, Mastropietro S, Borriello A and della Ragione F (1998) Differential expression of cyclin dependent kinase inhibitor genes (CDKN2A, CDKN2B) in human neuroblastoma: significant correlation between CDKN2B expression and advanced stage III and IV. *Pediatric Res* **43**: 139–144

Kallioniemi A, Kallioniemi OP, Sudar D, Rutovitz D, Gray JW, Waldman F and Pinkel D (1992) Comparative genomic hybridization for molecular cytogenetic analysis of solid tumors. *Science* **30**: 818–821

Lastowska M, Nacheva E, McGuckin A, Curtis A, Grace C, Pearson ADJ and Bown N (1997) Comparative genomic hybridization study of primary neuroblastoma tumors. *Genes Chromosom Cancer* **18**: 162–169

Lastowska M, Van Roy N, Bown N, Speleman F, Lunec J, Strachan T, Pearson ADJ and Jackson MS (1998) Molecular cytogenetic delineation of 17q translocation breakpoints in neuroblastoma cells lines. *Genes Chromosom Cancer* **23**: 116–122

Lo Cunsolo C, Iolascon A, Cavazzana A, Cusano R, Strigini P, Mazzocco K, Giordani L, Massimo L, De Bernardi B, Conte M and Tonini GP (1999) Neuroblastoma in two siblings supports the role of chromosome 1p36 in tumor development. *Cancer Genet and Cytogenet* **109**: 126–130

Meddeb M, Danglot G, Chudoba I, Venuat AM, Benard J, Avet-Loiseau H, Vasseur B, Le Paslier D, Terrier-Lacombe MJ, Hartmann O and Bernheim (1996) Additional copies of a 25 Mb chromosomal region originating from 17q23.1–17 qter are present in 90% of high-grade neuroblastomas. *Genes Chromosom Cancer* **17**: 156–165

Perri P, Pession A, Mazzocco K, Strigini P, Iolascon A, Basso G and Tonini GP (1996) Peculiar allelotype associated with susceptibility to neuroblastoma. *Genes Chromosom Cancer* **17**: 60–63

Peter M, Michon J, Vielh P, Neuenschwander S, Nakamura Y, Sonsino E, Zucker JM, Vergnaud G, Thomas G and Delattre O (1992) PCR assay for chromosome 1p deletion in small neuroblastoma samples. *Int J Cancer* **52**: 544–548

Plantaz D, Mohapatra G, Matthay KK, Pellarin M, Seeger RC and Feuerstein BG (1997) Gain of chromosome 17 is the most frequent abnormality detected in neuroblastoma by comparative genomic hybridization. *Am J Pathol* **50**: 81–89

Seeger RC, Brodeur GM, Sather H, Dalton A, Siege SE, Wong KY and Hammond D (1985) Association of multiple copies of the N-myc oncogene with rapid progression of neuroblastomas. *N Engl J Med* **313**: 1111–1116

Shimada H, Chatten J, Newton WA Jr, Sachs N, Hamoudi AB, Chiba T, Marsden HB and Misugi K (1984) Histopathologic prognostic factors in neuroblastic tumors: definition of subtypes of ganglioneuroblastoma and an age-linked classification of neuroblastomas. *J Natl Cancer Inst* **73**: 405–416

Takita J, Hayashi Y, Kohno T, Shiseki M, Yamaguchi N, Hanada R, Yamamoto K and Yokata J (1995) Allelotype of neuroblastoma. *Oncogene* **11**: 1829–1834

Tonini GP, Boni L, Pession A, Rogers D, Iolascon A, Basso G, Cordero di Montezemolo L, Casale F, Pession A, Perri P, Mazzocco K, Scaruffi P, Lo Cunsolo C, Marchese N, Milanaccio C, Conte M, Bruzzi P and De Bernardi B (1997) MYCN oncogene amplification in neuroblastoma is associated with worse prognosis, except in stage 4s. The Italian experience with 295 children. *J Clin Oncol* **15**: 85–93

Vandesompele J, Van Roy N, Van Gele M, Laureys G, Ambros P, Heimann P, Devalck C, Schuuring E, Brock P, Otten J, Gyselinck J, De Paepe A and Speleman F (1998) Genetic heterogeneity of neuroblastoma studied by comparative genomic hybridization. *Genes Chromosom Cancer* **23**: 141–152

Van Gele M, Van Roy N, Jauch A, Laureys G, Benoit Y, Schelfhout V, De Potter CR, Brock P, Uyttebroeck A, Sciot R, Schuuring E, Versteeg R and Speleman F (1997) Sensitive and reliable detection of genomic imbalances in human neuroblastomas using comparative genomic hybridisation analysis. *Eur J Cancer* **33**: 1979–1982

Van Roy N, Laureys G, Van Gele M, Opdenakker G, Miura R, van der Drift P, Chan A, Versteeg R and Speleman F (1997) Analysis of 1;17 translocation breakpoints in neuroblastoma: implications for mapping of neuroblastoma genes. *Eur J Cancer* **33**: 1974–1978

Wimmer K, Zhu XX, Lamb BJ, Kuick R, Ambros PF, Kovar H, Thoraval D, Motyka S, Alberts JR and Hanash SM (1999) Co-amplification of a novel gene, NAG, with the N-myc gene in neuroblastoma. *Oncogene* **18**: 233–238

 *© 2000 Cancer Research Campaign*
